# “Smart” Continuous Glucose Monitoring Sensors: On-Line Signal Processing Issues

**DOI:** 10.3390/s100706751

**Published:** 2010-07-12

**Authors:** Giovanni Sparacino, Andrea Facchinetti, Claudio Cobelli

**Affiliations:** Department of Information Engineering, University of Padova, Via Gradenigo 6/B, 35131 Padova, Italy; E-Mails: gianni@dei.unipd.it (G.S.); andrea.facchinetti@dei.unipd.it (A.F.)

**Keywords:** diabetes, prediction, filtering, calibration, model, time-series

## Abstract

The availability of continuous glucose monitoring (CGM) sensors allows development of new strategies for the treatment of diabetes. In particular, from an on-line perspective, CGM sensors can become “smart” by providing them with algorithms able to generate alerts when glucose concentration is predicted to exceed the normal range thresholds. To do so, at least four important aspects have to be considered and dealt with on-line. First, the CGM data must be accurately calibrated. Then, CGM data need to be filtered in order to enhance their signal-to-noise ratio (SNR). Thirdly, predictions of future glucose concentration should be generated with suitable modeling methodologies. Finally, generation of alerts should be done by minimizing the risk of detecting false and missing true events. For these four challenges, several techniques, with various degrees of sophistication, have been proposed in the literature and are critically reviewed in this paper.

## 1. Introduction

The knowledge of glucose concentration in blood is a key aspect in the quantitative understanding of the glucose-insulin system and in the diagnosis and treatment of diabetes. The use of signal processing techniques on glucose data started some decades ago, when glucose time-series in a given individual could be obtained in laboratories from samples drawn in the blood at a sufficiently high rate. In particular, an important body of literature of the 80s and 90s employed not only linear (e.g., correlation and spectrum analysis, peak detection), but also nonlinear (e.g., approximate entropy) methods to investigate oscillations present in glucose (and insulin) time-series obtained, during hospital monitoring, by drawing blood samples every 10–15 min for up to 48 h [1–3]. At that time, long term (e.g., days or months) studies resorted to self-monitoring blood glucose (SMBG) data, *i.e.*, three-to-four samples per day obtained by the patient himself by using fingerstick glucose meters. The retrospective analysis of SMBG time-series was used by physicians, together with the information taken from the “patient’s diary” (e.g., insulin dosage, meals intake, physical exercise) and some glycaemic indexes (typically HbA1c), to assess glucose control and the effectiveness of a particular therapy [4–7].

New scenarios in diabetes treatment were presented in the last ten years, when minimally invasive continuous glucose monitoring (CGM) sensors, able to monitor glucose concentration continuously for several days, entered clinical research [8–15]. This calls for more advanced techniques for studying glucose time-series. For instance, new insights can be obtained by analyzing the dynamics of the glucose signal, see e.g., Rahaghi and Gough [16] for a review. From a more practical point of view, retrospective analysis of CGM in place of SMBG data can facilitate diabetes management in a given individual (see Clarke and Kovatchev [17] for a review of the available statistical tools). In addition, since CGM devices can provide glucose readings in real-time, new on-line applications, with a potentially great impact in the patient’s daily life, have become of great interest. For instance, CGM signals are a key component of the so-called artificial pancreas, a device conceived for Type 1 diabetic patients aimed at maintaining glucose concentration within safe ranges by infusing insulin subcutaneously via a pump under the control of a closed-loop algorithm (see Hovorka and Cobelli *et al.* for two recent reviews [18,19]). Another important on-line application of CGM sensors is the generation of alerts when glucose concentration is predicted to exceed the normal range thresholds [20]. These applications require that CGM sensors become “smart” by means of algorithms able to interpret glucose levels in real-time. Indeed, several CGM sensors already in the market have some kind of alert system on board, even if their performance is still not satisfactory. In order to properly generate hypo/hyperglycemic alerts, in fact, at least four important aspects have to be considered. First, CGM data need to be accurately calibrated. Then, CGM data need to be filtered in order to enhance their signal-to-noise ratio (SNR). Thirdly, prediction of future glucose concentrations should be generated with suitable modeling methodologies. Finally, generation of alerts should minimize the risk of detecting false/missing true events. For these four challenges, several techniques, with various degrees of sophistication, have been proposed in the literature. The main achievements in this area are critically reviewed in this paper. Notably, being exhaustiveness difficult to achieve and, in any case, beyond the scope of the present special issue, we encourage the reader to also make reference to other reviews, with content in great part complementary to ours, which have been published very recently [21]. Finally, we note that we will review only general aspects, *i.e.*, signal processing, while possible sensor-dependant sources of error, e.g., related to specific sensor physics, chemistry and electronics, are not addressed here.

## 2. Calibration

**Figure 1.**
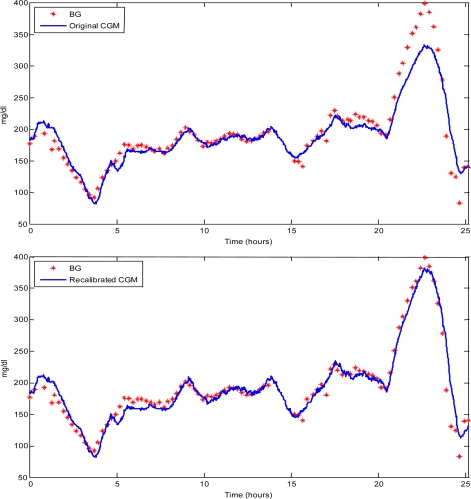
Representative type 1 diabetic subject. Top: BG references (stars) *vs.* original FreeStyle Navigator® CGM profile (continuous line). Bottom: BG references (stars) *vs.* CGM profile (blue line) profile recalibrated by the method of King *et al.* [32].

Most of the commercial minimally invasive CGM systems, e.g., and the CGMS^®^ (Medtronic Minimed Inc, Northridge, CA, USA) [22], the GlucoDay^®^ (Menarini Diagnostics, Florence, Italy) [23], the FreeStyle Navigator^®^ (Abbott Diabetes Care, Alameda, CA, USA) [24] and the Seven^®^ (DexCom Inc, San Diego, CA, USA) [25], exploit the glucose-oxidase principle, which requires that the measured current (e.g., in nA) be transformed into glucose levels (e.g., in mg/dL) by using one, or more, SMBG samples. This step is commonly referred to as a calibration [26]. Several studies have been performed in order to assess the influence of the number, accuracy, and temporal position of the reference SMBG samples, as well as by the trend of glucose concentration at their pick up times [27,28]. An important point related to calibration is that CGM sensors are placed in the subcutaneous tissue and thus they measure interstitial glucose (IG) rather than blood glucose (BG) concentration. In dynamic conditions, e.g., after a meal, IG and BG can be markedly different because of the existence of a BG-to-IG kinetics which, in the literature, has been described by a two-compartment model [29], *i.e.*,

(1) $$I\dot{G}(t)=-\frac{1}{\tau }\text{IG}(t)+\frac{g}{\tau }\text{BG}(t)$$

where g represents the static gain of the BG-to-IG system (which we can consider equal to 1, *i.e*., in steady state, the concentration of glucose in the blood and in the interstitium are equal) and τ is a time constant (which can vary between individuals). Equation (1) acts as a first order, linear, low-pass filter, and introduces a distortion, *i.e.*, attenuation in amplitude and phase delay, which is readily observable in Figure 1 (top panel). This figure shows a comparison, performed in a clinical study [30], on a type 1 diabetic subject, between and a FreeStyle Navigator® CGM profile and BG references collected every 15 min and measured in laboratory by YSI (Yellow Springs, OH, USA). Note that, in this and all the figures throughout the paper, time 0 is when data recording begins.

The existence of a BG-to-IG kinetics, however, is not able to explain some of the discrepancies which are evident along the y-axis, e.g., in the interval between 18–25 h. This difference is likely due to a change of behavior of the CGM sensor performance after its initial calibration.

The fact that CGM profiles can be affected by calibration problems can be critical in several applications, e.g., alert generation systems and artificial pancreas. For this reason, real-time “recalibration” of CGM data is desirable, where, for recalibration, we intend a step where the sensor output (in mg/dl) is processed by an algorithm (e.g., external to the device) in order to improve its accuracy. After recalibration, the difference between BG and CGM samples should be due to BG-to-IG kinetics only.

Several studies in the literature tried to cope with the recalibration problem, mostly retrospectively. A detailed study has been presented by the DirecNet Study Group [31], which analyzed the improvement in CGMS sensor accuracy by retrospectively modifying the number and timing of the calibration points. Results evidenced the fact that the timing of the calibration points is even more important than the number. In particular, performing calibrations during periods of relative glucose stability, *i.e.*, where the point-to-point difference due to the BG-to-IG kinetics is minimized, significantly improves the accuracy.

A more recent recalibration procedure, thought for an off-line application, is that presented by King *et al.* [32], which is based on the linear regression model:

(2) y = ax + b

where a and b are recalibration parameters which are determined by fitting them against a couple of BG and CGM pairs, *i.e.*, y and x in Equation (2), respectively, collected at the same time. For instance, once applied to the data of Figure 1 (top), where frequently collected references are available and used to fit Equation (2), this method produces the improved CGM profile shown in Figure 1 (continuous line, bottom panel). A more sophisticated recalibration approach was presented by Kuure-Kinsey *et al.* [33], where a dual-rate Kalman filter is used. The latter method exploits sparse SMBG measurements and can estimate a correction factor for the sensor gain in real-time.

None of the above recalibration algorithms consider explicitly the distortion introduced by BG-to-IG kinetics and the possibly time-varying behavior of sensor performance (the sensor gain is estimated only once for the entire monitoring). A first comprehensive description of the CGM measurement process is due to Knobbe and Buckingham [34]: BG-to-IG kinetics model was explicitly taken into account in order to reconstruct BG levels at continuous time from CGM measurements and an Extended Kalman Filter (EKF) approach was used to estimate the unknown variables. In a recent work of Facchinetti *et al.* [35], this idea was further developed in order to deal with recalibration. Reference SMBG samples were considered to be available on a sparse grid (e.g., four samples per day) and corrupted by additive measurement error, as:

(3) SMBG(k) = ϕ(k)BG(k) + e(k)

where φ(k) is a flag which is equal to 1 only at the times k when the SMBG sample is collected (the sampling interval is that of the CGM sensor). The measured CGM profile was modelled as:

(4) CGM(k) = (1 + α(k))IG(k) +v(k)

where IG(k) represents the “true”, perfectly calibrated, interstitial glucose level, v(k) is the random additive measurement error, and α(k) is a random multiplicative value which simulates the (possibly time-varying) deviation of the sensor gain from the ideal unit value [36]. IG(k) and BG(k) can be easily related by discretizing the continuous-time differential equation of Equation (1) (using a “population” value for τ). In order to estimate IG(k) and α(k) from the measured CGM(k) and SMBG(k) data, an *a priori* model is postulated in order to express the expected regularity of α(k) and BG(k), *i.e.*, their temporal profile is expected to be “smooth”. In particular, α(k) and BG(k) are modeled as the multiple integration of a white noise process (see Section 3 for more details on the formulation of *a priori* models of signal smoothness).

**Figure 2.**
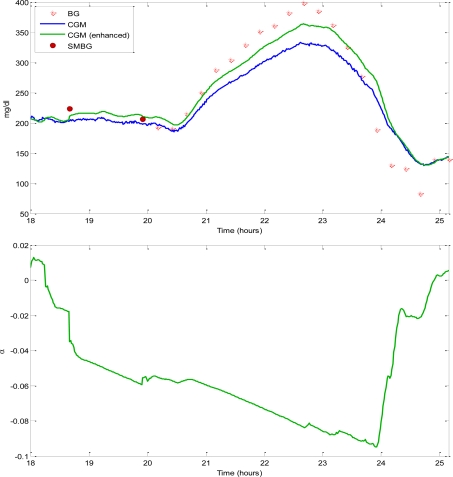
Representative type 1 diabetic subject (data taken from Kovatchev *et al.* [30]). Top: measured CGM (blue line), SMBG samples used for recalibration (copper circles), recalibrated CGM (green line), and other available SMBG samples plotted as reference (asterisks). Bottom: estimated deviation of the sensor gain from the unit value.

Under these assumptions, a nonlinear state-space nonlinear dynamic model can be obtained, where the unknown IG(k) and α(k) represent two of the unknown states, which can be estimated by EKF. An example on real data (collected in a type 1 diabetic subject) is shown in Figure 2, where only the two BG samples denoted by open bullets (top panel) are used to obtain, starting from the original CGM profile (blue line), the recalibrated CGM profile (green line). BG samples denoted by asterisks are not used for recalibration and are only left on the plot as a reference in order to graphically demonstrate the improvement of accuracy obtained by recalibration. In particular, after recalibration, the RMSE calculated between the CGM and the BG time-series decreases from 41 mg/dL to 32 mg/dL (−22%). The method is causal (thus potentially usable on line) and also provides the time course of the estimated gain deviation α(k) (bottom panel).

Realistic *in silico* simulations, obtained by using a simulator of the glucose-insulin system [37], showed that the recalibration method of Facchinetti *et al.* [35] improves the accuracy of CGM signals more than both the methods of King *et al.* [32] and Knobbe and Buckingham [34]. However, in Facchinetti *et al.* [35] it is also stated that the nonlinearity of EKF can render some steps, e.g., initialization of covariance matrices, critical and can also require rather long “burn-in” intervals. These difficulties can be circumvented by simpler recalibration strategies like those of Guerra *et al.* [38] and Facchinetti *et al.* [39] which, by embedding a deconvolution procedure, are able to account for BG-to-IG kinetics equally well.

## 3. Denoising

**Figure 3.**
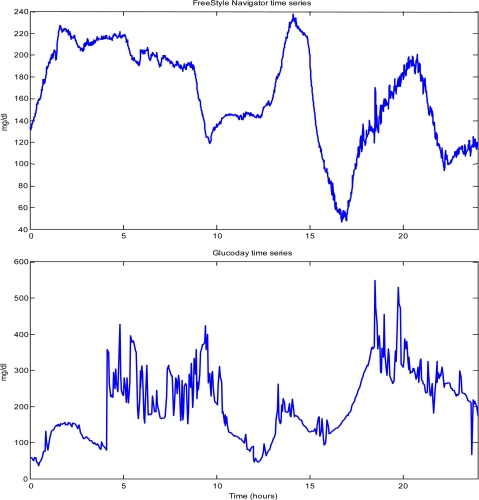
Two representative type 1 diabetic CGM time series. Top: FreeStyle Navigator® time series (1 min sampling), taken from Kovatchev *et al.* [30]. Bottom: GlucoDay® time series (3 min sampling) (taken from Maran *et al.* [44]).

In addition to calibration errors, the CGM signal is also corrupted by a random noise component. The noise typically dominates the true signal at high frequency and is usually considered to be additive:

(5) $$y_{k}\;=\;u_{k}\;+\;v_{k}$$

where u_k_ is the actual, but unknown, glucose level at time t_k_ and v_k_ is the random measurement error. The amount of noise corrupting the true glucose level is dependent on the sensor technology. For instance, some CGM profiles are more “stable” than others, as shown in Figure 3, where the time-series, measured for 24 h in two diabetic volunteers through the FreeStyle Navigator® (top panel) and the GlucoDay® (bottom panel), are displayed: in terms of Equation (5), the variance of the random process {v_k_} is lower in the first case (top panel). Moreover, a given sensor technology may behave differently in different subjects, e.g., the variance of {v_k_} may vary among subjects (compare the noise affecting the FreeStyle Navigator® time-series of Figure 3 and Figure 1). Another, less appreciated, ingredient of the noise affecting CGM data is that the SNR may even vary during a given recording. For instance, in the top panel of Figure 3, the noise variance in the time interval (18,24) is larger than in (8,18).

Given the expected spectral characteristics of noise, (causal) low-pass filtering [40] represents the most natural candidate to denoise CGM signals. One major problem in low-pass filtering is that, since signal and noise spectra normally overlap, it is not possible to remove noise v_k_ from the measured signal y_k_ without also distorting the true signal u_k_. In particular, distortion results in a delay affecting the estimate û_k_ with respect to the true u_k_: the more the filtering, the larger the delay. It is easily appreciated that having a consistently delayed, even if less noisy, CGM signal could severely limit its use in practice, e.g., for the generation of timely hypo-alerts. A clinically relevant issue is thus the establishment of a suitable compromise between the regularity of û_k_ and its delay with respect to the true u_k_. Understanding how denoising is done inside commercial CGM devices is often difficult, but evidence inferred from the patent literature indicate that nonlinear pre-filtering and moving-average (MA) filters are very often used [23–25].

Nonlinear pre-filtering like signal clipping, median filtering, and hard-bounding, are used in order to deal with e.g., spurious spikes which may occasionally occur during the monitoring (see e.g., the artifacts in the bottom panel of Figure 3, likely due to perturbations in the functioning of the microdialysis fiber of the GlucoDay® sensor induced by patient movements). For instance, hard-bounding considers, at any sampling time, the absolute difference between the present and the previous sample: if this value, relative to the sampling period, is higher than the maximum physiologically allowed, e.g., 4 mg/dL min, the last sample is corrected accordingly.

**Figure 4.**
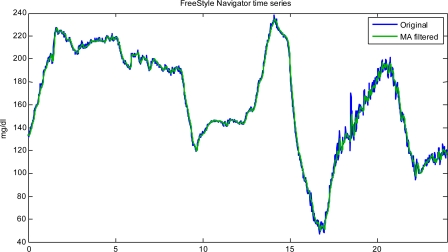

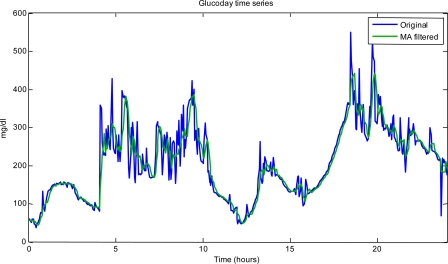
Same data as in Figure 3: original profile (blue) and outcome (green) of a MA filter with M = 5 and μ = 1.

As far as MA filtering is concerned, having fixed the so-called order M of the filter, the estimate of the signal at the k-th sampling time is a weighted sum of the last M measured samples:

(6) $${\hat{u}}_{k}=\frac{1}{\sum_{i=1}^{M}c_{i}}\left(c_{1}y_{k}+c_{2}y_{k-1}+...+c_{M}y_{k-M+1}\right)$$

where c_1,_ c_2,_… c_M_ are positive real numbers. The order M and the weights {c_k_} are crucial parameters to be tuned in the filter design. For instance, one choice is to make all the {c_k_} equal. Another frequent choice is to let c_i_ = μ^i^, where μ is a positive real (between 0 and 1) which, *de facto*, acts as a forgetting factor (the higher μ, the more slowly past data are forgotten). As far as M is concerned, increasing M in general produces a higher low-pass filtering. This can improve noise reduction, but can also result in a significant, and possibly unacceptable, signal distortion, e.g., the filtered sequence û_k_ cannot track fast changes present in the true sequence u_k_ and/or a significant delay is introduced. On the other hand, undersmoothing may leave the filtered profile hardly usable for making decisions such as whether or not an alert should be generated. Figure 4 displays these issues by applying the same MA filter (M = 5, with all the c_i_’s equal to 1) to the signals of Figure 3.

It is clear that any optimization made on order and weights of MA filtering cannot be directly transferred from one sensor to another. For instance, CGM time series observed with different sampling rates must be processed by filters with different parameters (even when they exhibit the same SNR, as it happens in some portions of the recordings of Figure 4). Moreover, filter parameters should be tuned according to the SNR of the time series, e.g., the higher SNR, the flatter the filtering (and the smaller the resulting distortion/delay). How to precisely tune filter parameters in an automatic manner is, however, a difficult problem, especially for MA filters.

**Figure 5.**
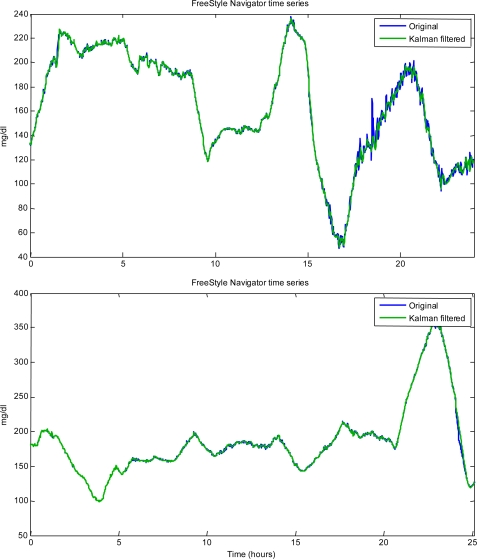
Application of the Kalman filtering method of Facchinetti *et al.* [43] to two FreeStyle Navigator® time-series exhibiting a different SNR (the green line is the filter output). Top panel: same time-series as in the top panel of Figures 3 and 4. Bottom panel: same time-series as in the top panel of Figure 1.

Some methods which can be used for denoising CGM signals with approaches more sophisticated than MA can be found in several works [33,34,41,42] More recently, the CGM denoising problem has been more comprehensively tackled by Facchinetti *et al.* [43], who have proposed an approach developed within a Bayesian estimation embedding and implemented by Kalman filtering (KF). The approach resorts to the idea that an “optimal” filter, *i.e.*, the filter which exhibits the best trade-off between noise reduction and signal distortion, can be designed by bringing the filter design problem within a Bayesian context [40]. Such an embedding requires the “*a priori*” second order statistical description of signal and noise (“*a priori*” here stands for “without having seen the measured data”). In detail, v_k_ is described as a white noise with zero mean and unknown variance σ^2^ (depending on the individual time series and, in general, time-varying). The unknown signal u_k_ is modeled as the realization of a stochastic process obtained by the cascade of a certain number of integrators driven by a zero-mean white noise process with (unknown) variance λ^2^ (these models were already mentioned in Section 2). This is a commonly-used, simple, but versatile way to give an a priori second order probabilistic description of a smooth time-series. For instance, when a single integrator is considered, the random-walk model is used:

(7) $$u_{k}\;=\;u_{k-1}\;+\;w_{k}$$

where {w_k_} is a sequence of white noise samples. Assuming a Gaussian setting, the “*a priori*” model of Equation (7) simply tells us that, given u_k–1_, then u_k_ will be with probability 99.7% in the range u_k–1_ ± 3λ, *i.e.*, the lower the λ value, the smoother the process {u_k_}. Here, λ^2^ is unknown and can be estimated, individually for each time-series, from the data {y_k_} together with σ^2^. This is possible using CGM data of a burn-in interval, by using a statistically-based smoothing criterion having a maximum likelihood interpretation. Once λ^2^ and σ^2^ have been estimated, the problem of extracting u_k_ from y_k_ (after the burn-in interval) can be numerically solved, in a computationally efficient manner, by resorting to the equations of the causal Kalman filter (KF). Therefore, a key feature of this method is that it can individualize the filter parameters λ^2^ and σ^2^, and hence the smoothing amount, according to the SNR of the specific CGM signal. Figure 5 shows an application of this method to the two FreeStyle Navigator® time-series of Figures 1 and 3.

The variance of the noise component in these two time-series is clearly different. The application of the denoising procedure provides estimates of σ^2^ equal, respectively from top to bottom, to 21.4 and 1.8 mg^2^/dL^2^, in line with the intuition that the first time-series is more noisy than the second one, and suggesting that a suitable amount of smoothing is introduced by the filtering method (for sake of completeness, the correspondent estimates of λ^2^ are 0.14 and 0.06 mg^2^/dL^2^, respectively). Interestingly, considering the top panel time-series, the time-lag introduced by Kalman filtering in Figure 5 is much smaller (1 *vs.* 2 min) than that obtained, for the same time-series, in Figure 4 (top panel) by using MA filtering.

In Facchinetti *et al.* [43] the performance of the new KF approach was quantitatively assessed on CGM GlucoDay® traces obtained from 24 subjects taken from Maran *et al.* [44]. Average results showed that, for comparable signal denoising, the delay introduced by KF is about 35% less than that obtained by MA. In addition, a comprehensive Monte Carlo study on synthetic data showed that the method is able to reliably estimate the SNR of the CGM time-series. In Facchinetti *et al.* [43], it was concluded that method is able to deal, automatically, with SNR inter-sensor and inter-individual variability. An additional refinement is the possibility of dealing with SNR intra-sensor variability. For instance, σ^2^ (and λ^2^) could be determined continuously on a sliding window [45] in order to track possible variations in SNR, e.g., due to possible deterioration of the sensor performance after several hours/days.

## 4. Prediction

A natural on-line application of CGM sensors is the prevention of hypo/hyperglycemic events. Only a few years after the appearance of CGM sensors in the market, some methods were proposed to generate alerts when the actual trend of the glucose concentration profile suggested that hypoglycemia was likely to occur within a short time. Such techniques are often termed projection methods. For instance, in Choleau *et al.* [46] an hypo-alert is generated when, on the basis of first-order linear extrapolation of glucose obtained from the last two/three samples, there is the risk that glucose concentration will cross the 70 mg/dL threshold within 20 min. Similar methodologies are conceivably implemented in commercial devices in order to timely detect dangerous trends [24].

An improvement can be obtained by generating hypo-/hyper-alerts on the basis of ahead-of-time prediction of glucose concentration, which can be computed from past CGM data and suitable time-series models. The possibility of making a short term prediction of glucose concentration exploiting its past history was originally suggested in Bremer and Gough [47], on the basis of preliminary results obtained from modeling blood glucose concentration data (not CGM), measured every 10 min in blood for up to 40 h, and using a prediction horizon (PH) of 10 min. Since then, several approaches have been proposed using CGM sensor data and a larger, and more clinically significant, PH. For instance, in Sparacino *et al.* [48], two simple low-order (time-varying) time-series models were tested in order to assess the possibility to predict glucose concentration, at a PH equal to 30 or 45 min, from CGM time-series. In particular, the glucose time-series was described, locally, by a first-order polynomial:

(8) $$u_{i}\;=\;{\alpha t}_{i}\;+\;\beta$$

or by an auto-regressive (AR) model of first-order, corresponding to the following time-domain difference equation:

(9) $$u_{i}\;=\;{\text{au}}_{i}{}_{-1}+w_{i}$$

In Equation (9), i = 1, 2, …n denotes the order of glucose samples collected till the n-th sampling time t_n_ and {w_i_} is a random white noise process with zero mean and variance equal to σ^2^. Both models, Equations (8) and (9), are simple, but their parameters are adaptive, and are re-identified at each sampling time in order to track changes in the signal dynamics. Formally, letting **θ** to denote the vector of the parameters of the model employed to describe the glucose time-series, at each sampling time t_n_, a new value of **θ** is determined by fitting past glucose data u_n_, u_n−1_, u_n−2_, ... by weighted linear least squares. Once **θ** is determined, the model is used to calculate the prediction of glucose level Q steps ahead (where Q is such that Q·T_s_ = PH, where T_s_ is the sensor sampling period). For instance, in the case of Equation (9):

(10) $${\hat{u}}_{n+Q}\;=\;a^{Q}u_{n}$$

The necessity of having a time-varying **θ** is obvious in the model of Equation (8). For other models, e.g., AR models, the use of a time-invariant **θ**, e.g., identified in a burn-in interval, could produce inaccurate predictions because of the nonstationarity of CGM time-series. This is particularly true in the case of a low-order model (which can capture signal dynamics only in a limited time-interval), so a time-varying modeling strategy is in order. In determining the model parameters **θ** at a given time, all the past data can participate, possibly with different relative weights. A typical choice is to employ exponential weighting, *i.e.*, μ^k^ is the weight of the sample taken k instants before the actual sampling time, with μ, taken in the range (0,1), acting as forgetting factor. If a forgetting factor is not used (which is equivalent to letting μ = 1), glucose samples collected tens of hours, if not days, before the actual sampling time would influence prediction, with a possible deterioration of the algorithm capability to promptly track changes in the signal, in particular those due to perturbations, e.g., meals. From an algorithmic point of view, recursive least squares (RLS) implementations are possible in order to estimate the unknown model parameters **θ** in a computationally efficient manner.

**Figure 6.**
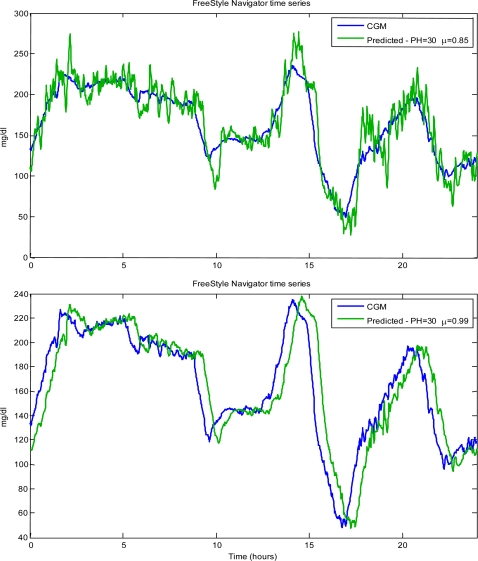
FreeStyle Navigator® time-series in a type 1 diabetic subject taken from Kovatchev *et al.* [30]. Original (blue line) *vs.* predicted (green line) CGM time-series by using an AR model of order 1 with PH = 30 and μ = 0.85 (too small) and μ = 0.99 (too large).

Figure 6 shows the original (solid) *vs.* the predicted (thin line) time-series for a representative type 1 diabetic subject. The predicted time-series were obtained using the AR model of order 1 with PH = 30 and, respectively from top to bottom, μ = 0.85 and 0.99. As apparent from the figure, the predicted profile is unavoidably affected by error: in particular, it is delayed and much more noisy than the original one.

Assessing how much such a kind of predicted profiles can be useful for the prevention hypo-/hyper-glycemia events is critical, but crucial. A straightforward index which could be considered, especially for comparing the relative performance of different models (e.g., polynomial *vs.* AR for different values of μ or PH) is the mean square prediction error (MSPE), which can be used to measure how close the predicted profile is to the original one. However, in a prediction context, MSPE is of limited use, e.g., a very unstable prediction profile may exhibit a smaller MSPE than a more regular prediction profile which could be safer for alert generation. Therefore, more specific indexes must be considered [48].

A useful index is the energy of the second order differences of the predicted profile (ESOD), which reflects the presence of spurious oscillations in the predicted profile (oscillations are obviously undesirable, since they can facilitate the generation of false hypo-/hyper-alerts). Another index is the delay of the predicted profile with respect to the original curve. In fact, the difference between PH and this delay represents a measure of the “gain in time” for alert generation obtained thanks to the use of predicted, instead of measured, CGM time-series. Notably, a delay of the prediction profile comparable to PH (or larger) would make the prediction profile useless in practice. All the above indexes are useful in assessing CGM time-series prediction algorithms. Of note is that, they are all dependent on the chosen PH and forgetting factor μ. For instance, as well visible in Figure 6, a larger μ (bottom panel) provides a more stable prediction profile (smaller ESOD), at the cost of loosing the ability to promptly track changes in the glucose trend (larger delay and MSPE).

**Figure 7.**
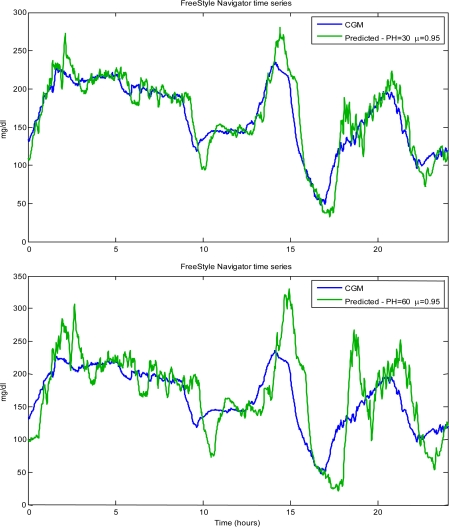
Same data of Figure 6: Original (blue) *vs.* predicted (green) profiles. Top panel: μ = 0.95 and PH = 30. Bottom panel: same μ as in top panel, but PH = 60.

Figure 7 (top panel) displays the predicted profile for the same time-series of Figure 6 obtained with PH = 30 but with μ = 0.95, a value which, retrospectively, realized a suitable compromise among the above described merit criteria. The role of μ is clear, but PH also plays a crucial role in any prediction algorithm. Obviously, one would like to have a PH as large as possible. However, an increase of PH causes a larger delay, and thus a larger prediction error, and wider oscillations in the predicted profiles (larger MSPE and ESOD) which may affect specificity in alert generation due to larger prediction errors. For instance, Figure 7 (bottom panel) illustrates the performance of the same algorithm used in Figure 7 (top panel), with the same μ = 0.95, when the PH is set to 60 min. Therefore, also the maximum allowable PH should be a matter of investigation. In general, the maximum allowable PH has to reflect both SNR and sampling rate (e.g., the higher SNR and/or the sampling rate, the higher the maximum allowable PH). We strongly encourage that the optimization of μ and PH be addressed simultaneously and within the same framework.

The problem of assessing the performance of a prediction algorithm has been presented by making reference to a specific prediction model, but it is completely general. As a matter of fact, how to optimally design a prediction algorithm for CGM data, e.g., model structure, order, prediction horizon, forgetting factor, is still an open issue. The work by Zanderigo *et al.* [49] considered the possibility of using Continuous Glucose Error-Grid Analysis (CG-EGA) [30] to compare the relative performance of different prediction algorithms and parameters from a clinical point of view. Another possibility is to develop new indexes able to simultaneously take into account all the above cited ingredients.

Another work using a low-order linear time-series modeling to predict CGM is that of Eren-Oruklu *et al.* [50]. In this study, AR of order 3 and AR moving average (ARMA) of order (3,1) prediction algorithms were developed and the identification of model parameters was performed by using a RLS implementation applying a forgetting factor μ. In addition, the RLS has been integrated with a change detection method, which decreases μ to a smaller value when a persistent change in model parameters is detected. This modification allows to rapidly capture glycaemic disturbances. The assessment of prediction performance has been performed by using as indexes the relative absolute difference between predicted and original time-series, the sum of squares of glucose prediction error and CG–EGA. Results were satisfactory in terms of accuracy, but no estimates of the prediction delays were reported.

In the approaches above, CGM time-series are described by a model with fixed structure, minimum complexity, but with time-varying parameters which, at each sampling time, are re-adjusted on the basis of the newly collected glucose sample. Since the model has to describe the time-series only “locally”, its complexity can be kept modest, a crucial aspect for using prediction algorithms in real-time. Reifman *et al.* [51] employed a different approach, which was usable only in nine iSense CGM time-series (1 min sampling), out of 15 measured. This approach exploited a high-order AR model (order 10):

(11) $$u_{n}=\sum_{k=1}^{10}a_{k}u_{n-k}+w_{n}$$

where u_n_ is the glucose samples collected at time t_n_ and w_n_ is a random white noise process. The model was first fitted, in each subject, in a burn-in interval and then used within the prediction algorithm for the rest of the time-series. A price to be paid for this choice lies in the increased model complexity which, in turn, requires the use of a rather long burn-in interval (about 2,000 samples, nearly 36 h). Moreover, given the high number of AR parameters to be estimated, the time-series model is overly sensitive to noise. Indeed, a “regularization constraint” was placed on the AR coefficients in order to decrease their sensitivity to the data. In Reifman *et al.* [51], the possibility of using a time-invariant AR “population” model was also suggested, and further developed in a successive study [52], even if the effect of the likely prediction distortion due to the suboptimality introduced by the use of a “population” model is critical (e.g., greater risk of generating false positives and negatives). The same team recently published also a paper where the same prediction modeling machinery is used to assess the relative importance and predictive power of different frequency bands of glucose signals [53]. Results suggest that, for short term prediction, no benefits are expected from the inclusion in AR models of information concerning meals and insulin intakes.

In Palerm and Bequette [54] a stochastic nonparametric approach was proposed. The approach was applied to 13 data sets of CGMS® data (5 min sampling) during a hypoglycemic clamp (4 h data observation). The idea of the method is to exploit the available a priori information on the smoothness of CGM signal, formalized through a stochastic model including the multiple integration of a white noise process. After having placed the problem in a state-space setting, e.g.:

(12) $$\left\{ \begin{array}{l} x_{k+1}=\Phi x_{k}+w_{k}\\ y_{k}=Cx_{k}+v_{k} \end{array}\right.$$

where x_k_ is the state space vector at time k, y_k_ is the measurement vector, Φ and C are transition matrices, and v_k_ and w_k_ are state and measurement noise vectors, the Kalman methodology is used to predict glucose level after a given PH. Three different PH values were tested, *i.e.*, PH = 10, 20, and 30 min. The parameters of the Kalman filter were empirically determined, retrospectively, in order to “maximize” sensitivity and specificity. The authors reported that the prediction performance, in this well-controlled hypoglycaemic clamp situation, was satisfactory in terms of sensitivity and specificity, but, again, no estimates of the delays were reported.

Recently, an artificial neural network model (NNM) has also been applied in the prediction of glucose concentrations using CGM data. The use of NNM for glucose prediction is appealing because it could facilitate the exploitation of information on meals and insulin administrations. In Pappada *et al.* [55] a NNM was developed and trained both with data from the CGMS® and other information manually recorded by the patient in an electronic diary (*i.e.*, SMBG values, insulin dosages, carbohydrate intakes, hypo-/hyper-glycemic symptoms, lifestyle activities). Even if the trend prediction is acceptable, the NNM underestimates extreme hyperglycemic values and overestimates hypoglycemic values, making the method likely of limited use in real-time applications. Finally, in a recent work of Pérez-Gandía *et al.* [56], a real-time prediction algorithm based on NNM, where the model was trained by using only CGM data as inputs, was developed. A preliminary assessment of the method on six Freestyle Navigator® and on nine CGMS® time-series provided encouraging results.

## 5. Alert generation

A critical problem is the generation of alerts, see Heise *et al.* [57] for requirements and concepts. Real-time alert systems were available e.g., on the Glucowatch® G2 BiograPHer (Cygnus, Redwood City, CA, USA), (no longer available in the market) [58] and on the Guardian® (Medtronic Minimed, Northridge, CA, USA) [59]. Both systems generate alerts on the basis of the comparison of the actual glucose level and a pre-selected threshold. However, the efficacy of these systems is controversial [60]. In particular, some studies evidenced the unacceptably high percentage of false alerts, which, for allowing sufficient sensitivity, often results higher than 50% [20,61]. In addition, these systems cannot avoid the event, because they generate the alert when the event occurs. In order to overcome this limitation, some devices now perform a trend analysis, indicating direction and rate of change of glucose, in order to provide the patient with an early warning. However, to the best of our knowledge, no large scale studies have quantitatively documented in peer-reviewed articles the benefit of this procedure.

**Figure 8.**
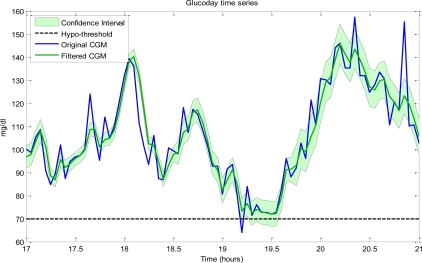
Risk of generating a false hypo-alert from the original CGM profile (blue line) at time 19.2 mitigated by employing an on-line filtered profile (green line) together with its confidence interval (shaded area).

Generating alerts accurately is difficult, because CGM data are often inaccurate (*i.e.*, biased) due to calibration problems and always uncertain (*i.e.*, noisy). The statistical basis behind the generation of alerts should thus be put on more solid grounds by considering, in addition to a trivial threshold comparison, the uncertainty of the data, which should be estimated in real-time in a statistical setting to determine, in real-time, the actual, possibly time-varying, SNR. To better illustrate this issue, in Figure 8 we illustrate a CGM profile (blue line) and its filtered version (green line) obtained in real time by the method of Facchinetti *et al.* [43]. Thanks to its Bayesian setting, this method also provides the confidence interval (shaded area) of the filtered CGM profile. Remarkably, the higher the noise affecting a given portion of data, the larger the confidence interval of the denoised profile. Notably, if the original CGM profile (blue line) is used to generate alerts by comparing the actual glucose level with a threshold (dashed black horizontal line), there is a significant risk of generating false alerts (see e.g., the event at time 19.2). The risk can be mitigated if the filtered profile is considered in the decision criterion, together with the portion of the confidence interval which exceeds the threshold [45]. Other possibilities can resort to prediction methods, but, at the present time, no results are available. However, generating alerts from the prediction is even more difficult than from the actual CGM data, given the fact that the ahead-of-time predicted profile is less stable than the original one (see Figures 6 and 7). This exacerbates the problem of avoiding false alerts. Rendering the predicted profile less sensitive to noise, e.g., by reducing the PH or by increasing the forgetting factor, obviously affects sensitivity and reduces the “temporal gain” of prediction and, thus, the practical advantage of using the predicted in place of the original CGM profile. Needless to say, strategies to generate alerts should take into account not only the nominal predicted glucose level, but also its uncertainty. Of note is that the higher the PH, the larger the confidence interval of the predicted glucose level.

## 6. Conclusions

CGM sensors allow the development of new strategies for the treatment of diabetes. In this contribution, we have considered four specific issues which are crucial for a “smart” real-time application of CGM sensors, in both open-loop (alert generators) and closed-loop (artificial pancreas) systems: (re)calibration for enhancing the accuracy of CGM signals, filtering for the enhancement of the SNR, ahead-of-time prediction, and generation of hypo/hyper-alerts. The main achievements of the literature, and also some open issues, have been discussed.

## References

[1] O'Meara NM, Sturis J, Van Cauter E, Polonsky KS (1993). Lack of control by glucose of ultradian insulin secretory oscillations in impaired glucose tolerance and in non-insulin-dependent diabetes mellitus. J. Clin. Invest.

[2] Simon C, Brandenberger G, Follenius M (1987). Ultradian oscillations of plasma glucose, insulin, and C-peptide in man during continuous enteral nutrition. J. Clin. Endocrinol Metab.

[3] Hollingdal M, Juhl CB, Pincus SM, Sturis J, Veldhuis JD, Polonsky KS, Pørksen N, Schmitz O (2000). Failure of physiological plasma glucose excursions to entrain high-frequency pulsatile insulin secretion in type 2 diabetes. Diabetes.

[4] Magni P, Bellazzi R (2006). A stochastic model to assess the variability of blood glucose time series in diabetic patients self-monitoring. IEEE Trans. Biomed. Eng.

[5] Deutsch T, Lehmann ED, Carson ER, Roudsari AV, Hopkins KD, Sönksen PH (1994). Time series analysis and control of blood glucose levels in diabetic patients. Comput. Methods Programs Biomed.

[6] Lehmann ED (1997). Application of computers in clinical diabetes care. Diab. Nutr. Metab.

[7] Kovatchev BP, Otto E, Cox D, Gonder-Frederick L, Clarke W (2006). Evaluation of a new measure of blood glucose variability in diabetes. Diabetes Care.

[8] Klonoff DC (2005). Continuous glucose monitoring: roadmap for 21st century diabetes therapy. Diabetes Care.

[9] Skyler JS (2009). Continuous glucose monitoring: an overview of its development. Diabetes Technol. Ther.

[10] Buckingham B (2008). Clinical overview of continuous glucose monitoring. J. Diabetes Sci. Technol.

[11] Deiss D, Bolinder J, Riveline J, Battelino T, Bosi E, Tubiana-Rufi N, Kerr D, PHillip M (2006). Improved glycemic control in poorly controlled patients with type 1 diabetes using real-time continuous glucose monitoring. Diabetes Care.

[12] Garg K, Zisser H, Schwartz S, Bailey T, Kaplan R, Ellis S, Jovanovic L (2006). Improvement in glycemic excursions with a transcutaneous, real-time continuous glucose sensor. Diabetes Care.

[13] The Juvenile Diabetes Research Foundation Continuous Glucose Monitoring Study Group (2008). Continuous glucose monitoring and intensive treatment of type 1 diabetes. N. Engl. J. Med.

[14] De Block C, Vertommen J, Manuel-y-Keenoy B, Van Gaal L (2008). Minimally-invasive and non-invasive continuous glucose monitoring systems: indications, advantages, limitations and clinical aspects. Curr. Diabetes Rev.

[15] Nichols JH, Klonoff DC (2007). The need for performance standards for continuous glucose monitors. J. Diabetes Sci. Technol.

[16] Rahaghi FN, Gough DA (2008). Blood glucose dynamics. Diabetes Technol. Ther.

[17] Clarke W, Kovatchev B (2009). Statistical tools to analyze continuous glucose monitor data. Diabetes Technol. Ther.

[18] Hovorka R (2008). The future of continuous glucose monitoring: closed loop. Curr. Diabetes Rev.

[19] Cobelli C, Dalla Man C, Sparacino G, Magni L, De Nicolao G, Kovatchev BP (2009). Diabetes: Models, Signals, and Control. IEEE Rev. Biomed. Eng.

[20] Buckingham B (2005). Hypoglycemia detection, and better yet, prevention, in pediatric patients. Diabetes Technol. Ther.

[21] Bequette BW (2010). Continuous Glucose Monitoring: Real-Time Algorithms for Calibration, Filtering, and Alarms. J. Diabetes Sci. Technol.

[22] Mastrototaro JJ, Gross TM, Shin JJ (2002). Glucose monitor calibration methods. US Patent 6,424,847.

[23] Poscia A, Mascini M, Moscone D, Luzzana M, Caramenti G, Cremonesi P, Valgimigli F, Bongiovanni C, Varalli M (2003). A microdialysis technique for continuous subcutaneous glucose monitoring in diabetic patients (part 1). Biosens. Bioelectron.

[24] Feldman BJ, McGarraugh GV (2007). Method of calibrating an analyte measurement device, and associated methods, devices and systems. US Patent 7,299,082.

[25] Simpson P, Brister M, Wightlin M, Pryor J (2008). Dual electrode system for a continuous analyte sensor. Publication Number WO/2008/042918.

[26] Lodwig V, Heinemann L, Glucose Monitoring Study Group (2003). Continuous glucose monitoring with glucose sensors: calibration and assessment criteria. Diabetes Technol. Ther.

[27] Panteleon AE, Rebrin K, Steil GM (2003). The role of the independent variable to glucose sensor calibration. Diabetes Technol. Ther.

[28] Choleau C, Dokladal P, Klein JC, Ward WK, Wilson GS, Reach G (2002). Prevention of hypoglycemia using risk assessment with a continuous glucose monitoring system. Diabetes.

[29] Rebrin K, Steil GM, Van Antwerp WP (1999). Subcutaneous glucose predicts plasma glucose independent of insulin: implications for continuous monitoring. Am. J. Physiol.

[30] Kovatchev B, Gonder-Frederick LA, Cox DJ, Clarke WL (2004). Evaluating the accuracy of continuous glucose monitoring sensors: continuous glucose-error grid analysis illustrated by the TheraSense Freestyle Navigator data. Diabetes Care.

[31] Buckingham BA, Kollman C, Beck R, Kalajian A, Fiallo-Scharer R, Tansey MJ, Fox LA, Wilson DM, Weinzimer SA, Ruedy KJ, Tamborlane WV, Diabetes Research In Children Network (Direcnet) Study Group (2006). Evaluation of factors affecting CGMS calibration. Diabetes Technol. Ther.

[32] King C, Anderson SM, Breton M, Clarke WL, Kovatchev BP (2007). Modeling of calibration effectiveness and blood-to-interstitial glucose dynamics as potential confounders of the accuracy of continuous glucose sensors during hyperinsulinemic clamp. J. Diabetes Sci. Technol.

[33] Kuure-Kinsey M, Palerm CC, Bequette BW A dual-rate Kalman filter for continuous glucose monitoring.

[34] Knobbe EJ, Buckingham B (2005). The extended Kalman filter for continuous glucose monitoring. Diabetes Technol. Ther.

[35] Facchinetti A, Sparacino G, Cobelli C (2010). Enhanced accuracy of continuous glucose monitoring by online extended Kalman filtering. Diabetes Technol. Ther.

[36] Facchinetti A, Sparacino G, Cobelli C (2010). Modeling the error of continuous glucose monitoring sensor data: critical aspects discussed through simulation studies. J. Diabetes Sci. Technol.

[37] Dalla Man C, Rizza RA, Cobelli C (2007). Meal simulation model of the glucose-insulin system. IEEE Trans. Biomed. Eng.

[38] Guerra S, Facchinetti A, Sparacino G, De Nicolao G, Cobelli C Comparison of four methods for on-line calibration of CGM data.

[39] Facchinetti A, Guerra S, Sparacino G, De Nicolao G, Cobelli C (2009). Method to Recalibrate Continuous Glucose Monitoring Data On-Line. US Provisional Patent Application No. 61/257,288.

[40] Anderson BD, Moore JB (2005). Optimal Filtering.

[41] Chase JG, Hann CE, Jackson M, Lin J, Lotz T, Wong XW, Shaw GM (2006). Integral-based filtering of continuous glucose sensor measurements for glycaemic control in critical care. Comput. Methods Programs Biomed.

[42] Palerm CC, Willis JP, Desemone J, Bequette BW (2005). Hypoglycemia prediction and detection using optimal estimation. Diabetes Technol. Ther.

[43] Facchinetti A, Sparacino G, Cobelli C (2010). An online self-tunable method to denoise CGM sensor data. IEEE Trans. Biomed. Eng.

[44] Maran A, Crepaldi C, Tiengo A, Grassi G, Vitali E, Pagano G, Bistoni S, Calabrese G, Santeusanio F, Leonetti F, Ribaudo M, Di Mario U, Annuzzi G, Genovese S, Riccardi G, Previti M, Cucinotta D, Giorgino F, Bellomo A, Giorgino R, Poscia A, Varalli M (2002). Continuous subcutaneous glucose monitoring in diabetic patients: a multicenter analysis. Diabetes Care.

[45] Sparacino G, Facchinetti A, Cobelli C (2009). University of Padova. Method and device for processing glycemia level data by means of self-adaptive filtering, predicting the future glycemia level and generating alerts. Publication Number WO/2009/136372.

[46] Choleau C, Klein JC, Reach G, Aussedat B, Demaria-Pesce V, Wilson GS, Gifford R, Ward WK (2002). Calibration of a subcutaneous amperometric glucose sensor implanted for 7 days in diabetic patients. Part 2. Superiority of the one-point calibration method. Biosens. Bioelectron.

[47] Bremer T, Gough DA (1999). Is blood glucose predictable from previous values? A solicitation for data. Diabetes.

[48] Sparacino G, Zanderigo F, Corazza C, Maran A, Facchinetti A, Cobelli C (2007). Glucose concentration can be predicted ahead in time from continuous glucose monitoring sensor time-series. IEEE Trans. Biomed. Eng.

[49] Zanderigo F, Sparacino G, Kovatchev BP, Cobelli C (2007). Glucose prediction algorithms from continuous monitoring data: assessment of accuracy via continuous glucose—error grid analysis. J. Diabetes Sci. Technol.

[50] Eren-Oruklu M, Cinar A, Quinn L, Smith D (2009). Estimation of future glucose concentrations with subject-specific recursive linear models. Diabetes Technol. Ther.

[51] Reifman J, Rajaraman S, Gribok A, Ward WK (2007). Predictive monitoring for improved management of glucose levels. J. Diabetes Sci. Technol.

[52] Gani A, Gribok AV, Rajaraman S, Ward WK, Reifman J (2009). Predicting subcutaneous glucose concentration in humans: data-driven glucose modeling. IEEE Trans. Biomed. Eng.

[53] Lu Y, Gribok A, Ward K, Reifman J (2010). The importance of different frequency bands in predicting subcutaneous glucose concentration in type 1 diabetic patients. IEEE Trans Biomed Eng.

[54] Palerm CC, Bequette W (2007). Hypoglycemia detection and prediction using continuous glucose monitoring—a study on hypoglycemic clamp data. J. Diabetes Sci. Technol.

[55] Pappada SM, Cameron BD, Rosman PM (2008). Development of a neural network for prediction of glucose concentration in type I diabetes patients. J. Diabetes Sci. Technol.

[56] Pérez-Gandía C, Facchinetti A, Sparacino G, Cobelli C, Gómez EJ, Rigla M, de Leiva A, Hernando ME (2010). Artificial neural network algorithm for on-line glucose prediction from continuous glucose monitoring. Diabetes Technol. Ther.

[57] Heise T, Koschinsky T, Heinemann L, Lodwig V, Glucose monitoring study group (2003). Hypoglycemia warning signal and glucose sensors: requirements and concepts. Diabetes Technol. Ther.

[58] Pitzer KR, Desai S, Dunn T, Edelman S, Jayalakshmi Y, Kennedy J, Tamada JA, Potts RO (2001). Detection of hypoglycemia with the GlucoWatch biograPHer. Diabetes Care.

[59] Bode B, Gross K, Rikalo N, Schwartz S, Wahl T, Page C, Gross T, Mastrototaro J (2004). Alarms based on real-time sensor glucose values alert patients to hypo- and hyperglycemia: the guardian continuous monitoring system. Diabetes Technol. Ther.

[60] Klonoff DC (2004). The need for separate performance goals for glucose sensors in the hypoglycemic, normoglycemic, and hyperglycemic ranges. Diabetes Care.

[61] Ward WK (2004). The role of new technology in the early detection of hypoglycaemia. Diabetes Technol. Ther.

